# A Bayesian adaptive design for biomarker trials with linked treatments

**DOI:** 10.1038/bjc.2015.278

**Published:** 2015-08-11

**Authors:** James M S Wason, Jean E Abraham, Richard D Baird, Ioannis Gournaris, Anne-Laure Vallier, James D Brenton, Helena M Earl, Adrian P Mander

**Affiliations:** 1 MRC Biostatistics Unit Hub for Trials Methodology Research, Cambridge, UK; 2 Department of Oncology, University of Cambridge and NIHR Cambridge Biomedical Research Centre, Cambridge, UK; 3 Cambridge Breast Unit, Cambridge University Hospital NHS Foundation Trust, Cambridge, UK; 4 Cambridge Experimental Cancer Medicine Centre, Cambridge, UK; 5 Cancer Research UK Cambridge Institute, Li Ka Shing Centre, University of Cambridge, Cambridge, UK

**Keywords:** adaptive design, adaptive randomisation, Bayesian, biomarkers, phase II, stratified medicine

## Abstract

**Background:**

Response to treatments is highly heterogeneous in cancer. Increased availability of biomarkers and targeted treatments has led to the need for trial designs that efficiently test new treatments in biomarker-stratified patient subgroups.

**Methods:**

We propose a novel Bayesian adaptive randomisation (BAR) design for use in multi-arm phase II trials where biomarkers exist that are potentially predictive of a linked treatment's effect. The design is motivated in part by two phase II trials that are currently in development. The design starts by randomising patients to the control treatment or to experimental treatments that the biomarker profile suggests should be active. At interim analyses, data from treated patients are used to update the allocation probabilities. If the linked treatments are effective, the allocation remains high; if ineffective, the allocation changes over the course of the trial to unlinked treatments that are more effective.

**Results:**

Our proposed design has high power to detect treatment effects if the pairings of treatment with biomarker are correct, but also performs well when alternative pairings are true. The design is consistently more powerful than parallel-groups stratified trials.

**Conclusions:**

This BAR design is a powerful approach to use when there are pairings of biomarkers with treatments available for testing simultaneously.

Response to treatment is highly heterogeneous in many diseases, especially cancer. Some cancer treatments work extremely well in subgroups of patients and provide less benefit, or even harm, in other subgroups as exemplified by the interaction between KRAS mutation status and cetuximab (Jimeno *et al*, 2009) or panitumumab treatment in metastatic colorectal cancer (CRC; Peeters *et al*, 2013). The increasing availability of genetic and cancer genomic information means that it is now feasible for clinical trials to be based on genomic and proteomic information, that may stratify cancers into discrete disease subgroups which are also predictive for treatment response. To ensure high-quality information is available to the clinician, robust evidence must be gathered on the effect of treatment in these different subgroups. Development and validation of biomarker–treatment pairings is therefore particularly important.

Heterogeneity of response makes the traditional drug development and application process slow, costly and inefficient (Adams and Brantner, 2006; Yap *et al*, 2010). Clinical trials that focus on all patients often do not provide good evidence about differential response in subgroups (Stewart and Kurzrock, 2009). Instead, when treatments are expected to work only in subgroups, small enriched phase III trials are more efficient. To ensure that subgroups in phase III trials are suitably chosen, well-designed phase II trials are important.

The BATTLE (Kim *et al*, 2011), I-SPY2 (Barker *et al*, 2009) and FOCUS4 trials (Kaplan *et al*, 2013) are examples of trials using new methodology to simultaneously evaluate several experimental treatments and putative predictive biomarkers. The BATTLE and I-SPY2 trials use Bayesian adaptive randomisation (BAR) methodology to assign more patients to treatments that have performed well for similar patients that were previously recruited.

In the BATTLE trial, 255 heavily pretreated non-small cell lung cancer (NSCLC) patients were randomised to one of four molecular targeted therapies, according to a biomarker profile determined from fresh tumour biopsies. Following an initial equal-randomisation period, patients were adaptively randomised using the emerging data. The same design, with different drug combinations, is employed in the follow-on BATTLE-2 study (Papadimitrakopoulou *et al*, 2013).

The I-SPY 2 trial is being conducted in the pre-operative (neoadjuvant) breast cancer setting. Patients with potentially curable early stage breast cancer have a baseline tumour biopsy and biomarker panel assessment, then all receive standard pre-operative treatment based on weekly paclitaxel. Similar to the BATTLE studies, in the initial phase patients are randomised equally between standard and experimental arms. After an interim analysis of biomarker–treatment interactions, randomisation is adapted so that patient allocation is increased to treatment arms that show early signs of benefit.

The FOCUS4 trial in CRC uses group-sequential multi-arm multi-stage methodology. After 16 weeks of chemotherapy, patients are randomised to placebo or experimental treatments in parallel biomarker-determined cohorts. Within each cohort, stopping rules allow treatments to be dropped due to lack of benefit.

In this paper we describe and evaluate a novel design for use in phase II cancer trials with several biomarker groups and novel treatments. The design is proposed for use in two trials currently in development; one for high-risk neo-adjuvant breast cancer and one for platinum-resistant high-grade serous ovarian cancer (HGSOC). In both cases, patients will be categorised into subgroups using biomarkers. For each biomarker there is a linked treatment that, prior to the trial starting, is thought likely to be effective on patients who test positive for the biomarker. The design we describe combines trial methodology from BATTLE, I-SPY 2 and FOCUS4 with novel ideas. We propose using BAR methodology so that patient treatment allocation (by biomarker) can be moved away from ineffective linked treatments in a cost- and time-efficient way. Previously BAR has been shown to perform well in terms of power in comparison with designs without interim analyses (Lee *et al*, 2010, 2012), and have similar operating characteristics to group-sequential multi-arm multi-stage approach (Freidlin and Korn, 2013; Wason and Trippa, 2014). We elaborate further on the reasons we chose to use a BAR design rather than a group-sequential design in the discussion. Although designed with phase II oncology trials in mind, the methodology will be suitable for use in other disease areas where potential novel biomarkers and treatments pairings exist.

## Motivating example

As a motivating example for the methodology proposed in this paper, we describe the SMARTer trials (Systematic bioMarker-directed Adaptive Randomised Trials in breast and ovary cancer), currently in development by our group. This is a proposal for two independent multi-arm phase II trials to simultaneously test the effectiveness of new therapies and also refine biomarkers that are most likely to be the reliable predictors of treatment outcome. Each trial will focus on three pairs of predictive biomarkers and linked treatments, which will be compared with the control treatment of chemotherapy alone.

Our approach represents a slight perspective shift compared with studies such as BATTLE-2 and I-SPY 2. Both these studies start with a collection of drugs that are deemed interesting for further development in NSCLC and breast cancer, respectively, and try to identify if one or more from a group of preselected biomarkers can predict for clinical benefit. Conversely, we start with a biomarker-driven subgroup that prior knowledge suggests may represent a distinct disease subset. Examples include primary platinum-resistant HGSOC with CCNE1 amplification or PTEN loss (Etemadmoghadam *et al*, 2009; Cai *et al*, 2014). On the basis of preclinical and early clinical data we then match this biomarker with a treatment deemed likely to confer clinical benefit such as a proteasome inhibitor in CCNE1-amplified HGSOC (Etemadmoghadam *et al*, 2013). Targeting a subset of a particular cancer type with an identifiable driver mutation has led to remarkable successes in the past few years, exemplified by vemurafenib treatment of BRAF-mutated melanoma (Chapman *et al*, 2011) and crizotinib treatment of ALK-rearranged NSCLC (Kwak *et al*, 2010). However, it is evident that many cancers harbour multiple mutations, frequently targeting well annotated pathways, such as the MAPK and PTEN/PI3K/mTOR pathways, without a clear driver event. Efforts to target these pathways in unselected patient populations have so far proven largely unsuccessful. An advantage of our approach in this situation is that, even if the original biomarker–drug pairing (e.g. PTEN loss and a pan-PIK3CA inhibitor) does not show benefit, we still retain considerable power to detect a drug effect in one of the other biomarker-driven subsets (e.g., CCNE1 amplification) as detailed in the following sections.

The objective is to use the accumulating clinical trial data generated to identify suitable combinations of effective targeted treatment and linked predictive biomarker to take forward for testing in an enriched randomised phase III trial.

## Materials and methods

### Trial design

A maximum number of 350 patients are to be recruited during each trial over 3 years. The trial has a total of four interim analyses. The first is when 100 patients have been recruited, and the other three are equally spaced throughout the remainder of the trial, each taking place when the specified number of patients have been recruited. A final analysis is conducted after all patients have been assessed. Although the methodology can be applied to trials with any number of experimental treatments, we restrict attention only to three—labelled as T1, T2 and T3. A control treatment is also included at all stages of the trial. Each experimental treatment is linked with a biomarker—labelled as B1, B2 and B3. The pairings represent the most plausible subgroups in which the experimental treatments are likely to have a high treatment effect. That is, for a particular biomarker there is a paired treatment that is thought likely to be beneficial for patients who are positive for that biomarker. Patients may be positive for none, one, two or all three biomarkers.

At recruitment, a patient's tumour is tested to determine which biomarkers are positive. After testing, the patient is then randomised to a treatment as described in the next section. In the current design, the primary end points are binary: pathologic complete response for the neoadjuvant breast cancer study and 6-month progression-free survival (PFS) rate for HGSOC. However, similar techniques could be applied for time-to-event outcomes such as PFS or overall survival.

### Initial stage

Patients who are recruited before the first interim analysis are randomised equally between control and all experimental treatments that are linked to their tumour biomarkers. As an example, if a patient is positive for B1 and B3, they would be allocated with equal probability to control, T1 and T3. Patients who are negative for all biomarkers are still included in the trial, and are randomised equally between all treatments and control.

### Interim analyses and treatment allocation

The Supplementary material provides a full technical description of the procedure used to allocate patients to treatments after the first interim analysis. Here we provide a less technical overview of the approach.

The purpose of the interim analyses is to use the data gathered during the trial to update the allocation probabilities. This is done using a BAR procedure.

At each interim analysis, a Bayesian logistic regression model is fitted that models the probability of treatment success as a function of an intercept parameter, treatment effects, biomarker effects and interactions between biomarker and treatment.

The Bayesian model requires prior distributions for all parameters considered. For all parameters other than the interactions, uninformative prior distributions are used (specifically, a uniform distribution between −10 and 10). For the interaction parameters, we use informative priors for interactions between linked treatment–biomarker pairs, and uninformative for other interactions. The informative prior distribution used is a normal distribution with mean and variance 1. The prior mean was picked based on work in the Supplementary material.

The informative priors are used so that linked experimental treatments will be favoured at the interim analyses unless there is considerable evidence that an alternative treatment is superior.

From the Bayesian model, it is possible to find the posterior probabilities that each experimental treatment is superior to control for each biomarker profile. These probabilities are then used to update the allocation probabilities to each arm using a generalised version of the method used in Wason and Trippa (2014), described fully in the Supplementary material. Briefly, the allocation is set separately for every possible biomarker profile in proportion to the posterior probabilities from the Bayesian model; as the trial progresses, the increasing amount of evidence accumulated results in a potentially quite different allocation compared with the start. The allocation to the control treatment is set so that, for each possible biomarker profile, the number of patients allocated to the control is (on average) equal to the number of patients on the best-performing experimental treatment. This ensures that the power of the trial to detect significant differences between effective experimental treatments and the control treatment is maximised.

Given the posterior probability of each arm being better than control, calculated at the most recent interim analysis, the allocation only depends on the number of patients recruited to each arm and in particular does not require knowledge of the patient outcomes. Thus the allocation probability can be updated after each patient is recruited with a similar amount of administrative effort as a traditional randomised trial using stratified randomisation. Note, however, that the posterior probabilities are only updated at each interim analysis, as they require full knowledge of patient outcomes.

### Final analysis and hypothesis testing

The final analysis occurs after all patients have been recruited and assessed. A similar model to the one used at the interim analyses is used, except using a classical logistic regression instead of a Bayesian version. Although a Bayesian model could be used, we choose to use a non-Bayesian final analysis so that the informative prior distributions are only used to guide the adaptation at interim analyses, and the final analysis is only based on data gathered during the trial.

There are a total of 12 hypotheses that may be tested. Three correspond to the effect of an experimental treatment on patients who are positive for its linked biomarker (i.e., B1–T1, B2–T2 and B3–T3). A further six correspond to the effect of experimental treatments on patients who are positive for non-linked biomarkers (e.g., B1–T2, B1–T3 etc.). The remaining three hypotheses correspond to the effect of each experimental treatment on patients who are negative for every biomarker. These hypotheses are specified in terms of the parameters in the logistic regression (see Supplementary material).

Each hypothesis is rejected if the relevant Wald test statistic is above 1.5 (equivalent to a one-sided *P*-value of <0.067). This is chosen to control the linked-BAR design's total probability of making a type I error (known as the family-wise error rate (FWER)) at 0.4. As we are considering a phase II setting where significant results will be tested in an appropriately powered phase III trial, we do not aim to control the FWER at a stringent rate. In fact, stringent control of FWER is not recommended in these cases (Wason *et al*, 2014). Previous work has shown that a high FWER can be optimal when considering multi-arm phase II trials (Wason *et al*, 2013).

Any null hypotheses that are rejected will result in consideration of a phase III trial in the relevant sub-population. For example, if T1 is found to work well for B1-positive patients, then a phase III trial of T1 *vs* control in B1-positive patients will be considered. If multiple null hypotheses are rejected, then it may be that multiple subgroups are considered in the same phase III trial. We note that hypothesis testing results in this trial are non-binding and that other factors will be considered before starting a phase III trial.

### Delay

A factor that is present in most clinical trials is delay between recruitment of patients and observation of their response to treatment. In our case there is a 6-month delay. Delay causes loss of efficiency in adaptive designs because at interim analyses there will be patients who have been recruited but not yet assessed. Thus, when the posterior probabilities are updated, patients who are not yet assessed do not contribute information to this re-assessment. The loss in efficiency depends on the recruitment rate of the trial, as well as the delay. As an example, if a trial recruited all patients within 6 months, then no adaptive procedure would be possible as recruitment (and treatment assignment) would be finished by the time the first patient provided information on the effectiveness of treatments. We assume that accrual continues at a uniform rate throughout the trial and is not paused at interim analyses.

An advantage of BAR is that it naturally deals with fixed delays (Wason and Trippa, 2014). If a patient has been assessed, then they are included in the re-assessment of allocation probabilities; otherwise they are not.

### Alternative designs

In order to benchmark the power and ethical properties of the design proposed in this paper, we compare it with three alternative designs. These are:

#### Parallel-group-stratified phase II trials

In this design, no interim analyses are conducted. Patients are randomised to control or linked treatments only throughout the trial. To keep the designs comparable, we assume that patients who are negative for all biomarkers are included and randomised equally between all the treatments.

#### Non-linked BAR design

This is similar to the linked-BAR design proposed, with two important differences: (i) at the beginning, patients are randomised equally between all available treatments; (ii) the informative priors are not used at interim analyses (i.e., the linked treatments are not prioritised). This design is chosen to be similar to the design used for trials such as BATTLE (Kim *et al*, 2011). However, we do also use the improved procedure for setting the allocation to control.

#### Equal-randomisation design

In this design, no interim analyses are conducted. Patients are randomised equally between all treatments throughout the trial, regardless of their biomarker profile.

## Results

### Operating characteristics of design

In order to investigate the operating characteristics of the linked-BAR design, we assess it and the three comparison designs for eight different scenarios. In each scenario, response is simulated using a logistic model, but with parameter values depending on the scenario. In all cases, the biomarkers are assumed to have prevalence 0.3 and be independently assigned to patients. In addition to the eight scenarios, we considered different prevalence of the biomarkers and different recruitment rates of the trial, as these factors will both affect the power of the trial.

Table 1 summarises the various simulation scenarios we consider. In the Supplementary material, these scenarios are described in greater technical detail.

In each scenario, we simulated 2500 virtual trials for the two BAR designs, and 10 000 for the two other designs (the difference due to BAR simulations taking longer). Table 1 shows a summary of the results of this simulation study. The ‘Recommend any' column of scenario 1 shows the FWER of each design. Interestingly, the FWER is highest using the stratified design, with the linked-BAR having the lowest FWER. Subsequent power comparisons between the designs should take this into account.

When there is a positive interaction between a linked pair of treatment and biomarker (scenarios 2, 7), the parallel and linked-BAR designs have the highest power. The non-linked BAR design loses some power (5% in scenario 2 and 8% in scenario 7), although still performs fairly well.

When there is an interaction between an unlinked pair (scenario 3), the non-linked BAR approach performs best (74.0% power in scenario 3), as first stage patients are randomised to all treatments. The parallel trials procedure has poor power in that case (40.7% power), with the linked-BAR procedure intermediate between the two (67.6% power). These results indicate that the linked-BAR procedure has the highest power to detect a linked interaction, but still has fairly good power to detect non-linked interactions.

Although differences in power of between 5–10% may seem small, they are equivalent to a reduction in sample size of between 15–25%.

### Allocation

We examined how the probability of allocation between arms evolves as a BAR trial progresses. We considered two cases. In the first case we assumed that T1 provided a large positive benefit in B1-positive patients and that T2 provided a large negative effect in the same patients. In the second case, we reversed these, as in scenario 8 of Table 1. For 2500 simulation replicates, we kept track of the allocation probability of patients who were positive for B1 as the trial progressed.

**Figure 1:**
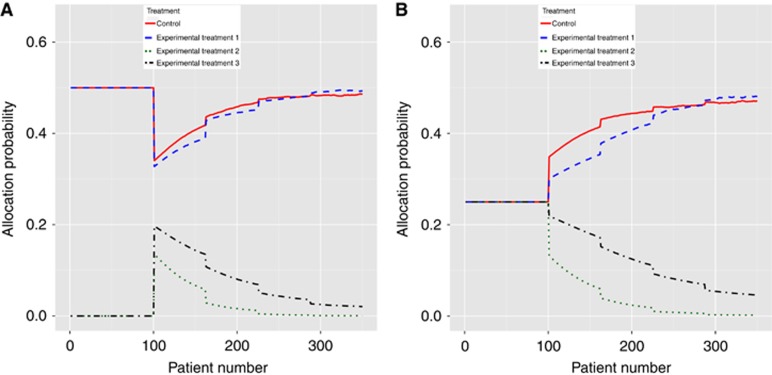
Mean allocation probability for: (**A**) linked-BAR design and (**B**) non-linked BAR design as trial progresses for a B1-positive patient when T1 provides benefit in B1-positive patients and T2 is detrimental in B1-positive patients. Lines represent the average over 2500 replicates.

Figure 1 shows the average allocation of the linked-BAR procedure (panel a) and the non-linked BAR procedure (panel b) in case 1. The linked-BAR procedure begins by randomising B1-positive patients equally between control and T1. This continues until the first interim analysis after 100 patients are recruited. After that the allocation to both falls slightly due to the BAR procedure being used. However, the average allocation then increases towards 0.5 as time goes on. As the allocation to T1 increases, so does the allocation to the control. The allocation to T2 and T3 decreases—notice that allocation to T2 falls more quickly due to it being inferior to control. A similar pattern is observed for the non-linked BAR design, although the initial allocation is equal to all arms. In addition, the allocation to T1 increases more slowly as informative priors are not used. Note also that the average allocation to the control group is higher than the average allocation to T1—this is because the control allocation is set to match the experimental treatment with the highest sample size, which by chance might not be T1 for a particular trial.

**Figure 2:**
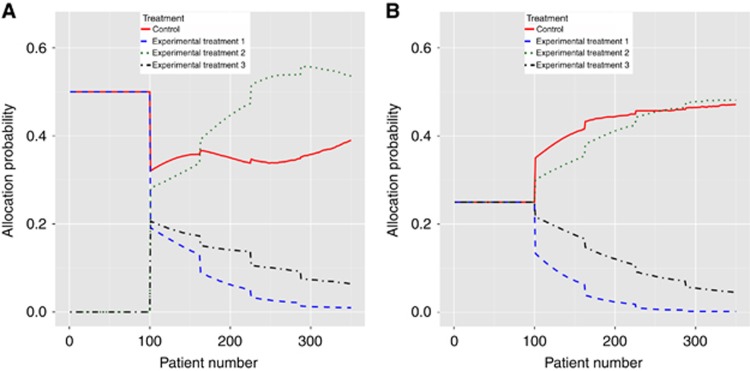
Mean allocation probability for: (**A**) linked-BAR design and (**B**) non-linked BAR design as trial progresses for a B1-positive patient when T2 provides benefit in B1-positive patients and T1 is detrimental in B1-positive patients. Lines represent the average over 2500 replicates.

Figure 2 shows the second case. In this case the linked-BAR approach starts allocating half of B1-positive patients to T1, but this drastically reduces after the first interim analysis and continues to decline as more data is gathered. The average allocation to T2, the superior treatment, increases as time goes on. The non-linked BAR approach performs better in this case because the allocation to T1 starts lower and drops more quickly due to a non-informative prior distribution.

### Varying the prevalence and recruitment rate

**Figure 3:**
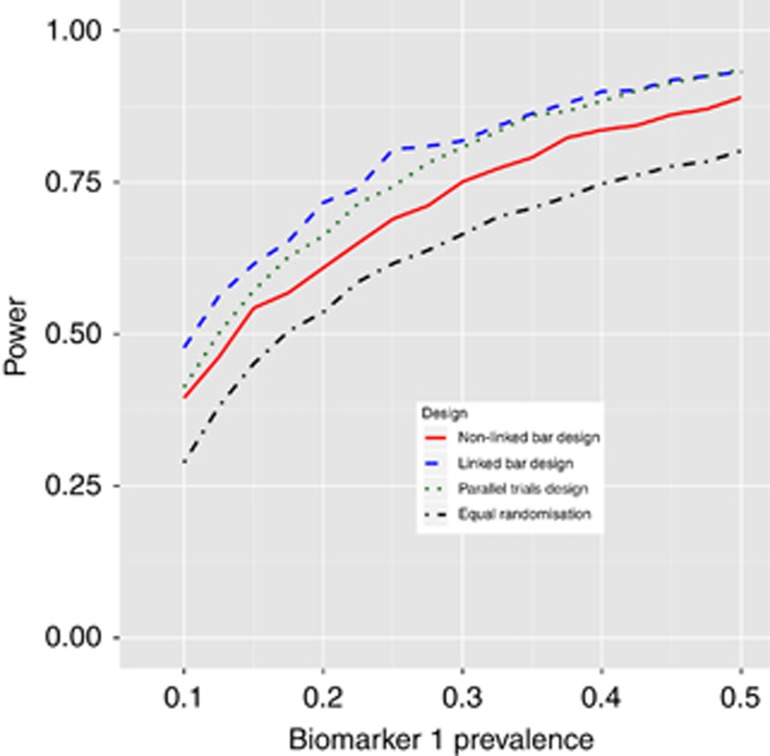
Power of the four designs to recommend T1 in B1-positive patients as prevalence of B1 changes under scenario 2 in Table 1.

We next examined the power of the four approaches to recommend T1 in B1-positive patients under scenario 2 as the prevalence of B1 changes between 0.1 and 0.5 in increments of 0.025. The results of this are shown in Figure 3. The power of all designs depends strongly on prevalence of B1. The linked-BAR design has below 50% power when the prevalence is 0.1, increasing to 90% power when the prevalence is 0.5.

Interestingly, the linked-BAR design generally has a slightly higher power compared with the parallel trials design. This is because of patients who are positive for multiple biomarkers; for example a patient who is positive for B1 and B2 will always be randomised equally between control, T1 and T2 in the parallel trials design, but will be more likely to be put on the more effective of T1 or T2 in the linked-BAR design.

We also investigated the sensitivity of the BAR methods to the recruitment rate. For simulation scenario 2, the power of both the non-linked BAR and linked-BAR designs remained at a similar level, with evidence of a 2–3% reduction for the non-linked BAR design as the recruitment rate increased from 7 patients per month to 11 patients per month (data not shown).

### Sensitivity analyses

In the Supplementary material, we provide results showing how the power of the linked-BAR design depends on the number of interim analyses and the prior mean used for the interactions between linked pairs of biomarker and treatment. The results show that five stages give sufficient power—increasing the number of stages further does not increase the power further. A prior mean of around 1 gives a good balance of power to detect linked treatments and non-linked treatments.

## Discussion

In this paper we have proposed a new method for use in phase II trials with several experimental treatments and biomarker subgroups. The new method, the linked-BAR design, is proposed for the situation where there are plausible pairings between the experimental treatments and biomarkers. That is, an experimental treatment is thought likely to have a positive treatment effect when patients are positive for the linked biomarker. We allow for these assumptions to be wrong by including interim analyses at which allocation probabilities are updated according to the relative effects of the treatments in different patient subgroups. We compared this design with the strategy of only randomising patients to linked treatments (i.e., not updating the allocation probabilities); an alternative, non-linked, BAR design which starts off randomising the patients to all available treatments and updates the allocation at interim analyses in a similar manner to the linked-BAR method; and a design that randomises patients equally throughout the trial regardless of their biomarker profile. The linked-BAR design has the highest power to detect a treatment effect for a linked biomarker–treatment pair and close to the highest power to detect a treatment effect between an unlinked pair. The design that randomises all patients equally throughout the trial generally performed poorly for testing hypotheses about the effect of treatments in biomarker subgroups. This indicates that a traditional trial design which ignores biomarker profiles of patients will give poor evidence for deciding which treatments work well in which biomarker groups.

The results show that there are situations where the non-linked BAR design is preferable to the linked-BAR design. For example, if there is no clear pairing between experimental treatments and biomarkers. In the case where the pairings have a biological rationale, or prior statistical evidence, then it is likely that the linked-BAR method will be more powerful. In pure power terms, the parallel trials design is only good when the prior linkages are highly likely to be true—its power suffers otherwise.

The choice of informative prior distribution in the linked-BAR design is important. A higher prior mean (meaning patients are more likely to be allocated to linked treatments), results in the power to detect treatment effects between linked treatment–biomarker pairs being higher than the parallel trials design. However, its power to detect non-linked pairs suffers considerably.

Different clinical scenarios can be accommodated with BAR methodology. First, if there is a hierarchy of biomarkers (e.g., a patient who is positive for B1 and B2 may be thought more likely to benefit from T1 than T2). Second, multiple treatments might be linked to a certain biomarker (or vice versa). In both cases, suitably changing the initial allocation and the prior distributions used for the parameters can accommodate the requirements. Third, if statistical interactions between biomarkers are plausible, then these can be included in the final analysis.

We base the planned interim analyses on when the recruitment milestones are met. It would be possible to instead plan analyses for when a pre-specified number of patients have outcome data available. This might well be preferable if the outcome had an unpredictable delay (e.g., when time-to-event end points are used), or when the recruitment rate could strongly vary during the trial.

Other biomarker-guided multi-arm trial designs have been proposed, for example I-SPY 2 (Barker *et al*, 2009) and FOCUS 4 (Kaplan *et al*, 2013). It is hard to directly compare our design with I-SPY 2 as there are few published details about the design available currently. It appears from the protocol and clinical paper (Barker *et al*, 2009) that the design is fairly similar to the non-linked BAR design we consider in this paper, except that different ‘clinically relevant' combinations of biomarkers are considered. The methodology we propose here (including the non-linked BAR design) has the power advantage that the control allocation is carefully set to match the experimental arm with highest sample size. On the other hand, the I-SPY 2 design allows early termination if there is enough evidence that an experimental treatment is superior and also uses longitudinal MRI measurements to augment the allocation procedure. Although we do not consider stopping rules here, they could easily be incorporated within the design we propose in a similar manner. In practice, if an allocation ratio for a treatment is below a threshold value (e.g., 5%) it would likely be set to 0.

Although we do not consider FOCUS4 comparable to the designs in this paper, due to it utilising a hierarchy of biomarkers, the question remains of whether a group-sequential design which specifies futility-stopping boundaries would be a superior choice to our BAR design. Previous work (Freidlin and Korn, 2013; Wason and Trippa, 2014) has shown that group-sequential and BAR designs have similar statistical operating characteristics in the context of a multi-arm trial without biomarker subgroups. The main advantage of the BAR approach is that it is not obvious how a group-sequential design would allow the linked treatments to be prioritised early in the trial whilst still allowing non-linked treatments to be allocated. A possibility would be to start by only randomising between control and linked treatments and then having a decision rule which would trigger randomising to non-linked treatments, but it is not clear how one should do this.

We have considered a trial design that only assesses the definitive end point. This is possible as the phase II end points we use are observed relatively quickly after recruitment. If the end points were longer-term then the methodology we propose in this paper would require a suitable intermediate end point that was known to be correlated with the final end point. Including intermediate end points in a BAR design has been considered by Berry *et al* (2010). We note also that the end point of progression at 6 months could be analysed as PFS by suitably changing the models used. We also could compare treatments for patients positive for multiple biomarkers using the parameters of the logistic regression.

Other relevant information, such as early progression from patients who have not yet reached 6-month follow-up, or safety and tolerability should also be considered at interim analyses. The allocation from the BAR procedure should be treated as a recommendation, and overridden as appropriate. For example, if all experimental arms are clearly superior to the control, the trial should likely be recommended for closing.

We have focused on the statistical properties of BAR designs in this paper, but there are non-statistical issues to be considered also. All types of adaptive designs will be accompanied by operational challenges (Gallo, 2006), for example, managing the randomisation and changing the allocation ratios after an interim analysis. It is also problematic that the number of patients to be assigned to each arm is not known in advance, as an adequate supply of drugs/treatments is required. Although this is an issue for all adaptive designs in biomarker-guided trials, BAR might experience greater problems as there is greater scope for deviation from equal randomisation. Another concern is potential investigator bias in enrolling patients if a particular biomarker–treatment combination is considered more attractive than others. This concern is minimised by the fact that, in our design, all patients receive at least standard-of-care chemotherapy and that the biomarker information is unlikely to be available outside of the trial setting.

We conclude that the novel linked-BAR trial methodology developed in this paper should provide a more cost-efficient trial design for future cancer phase II trials. We believe that the linked-BAR trial methodology will allow the most rapid and cost-efficient way of both matching and testing novel predictive biomarkers and new targeted treatments.

## Figures and Tables

**Table 1:** Description of simulation scenarios and simulation results for the eight scenarios

**Design**	**Recommend any**	**Recommend T1 in negative patients**	**Recommend T1 in B1-positive patients**	**Recommend T1 in B2-positive patients**	**Recommend T2 in B1-positive patients**
**Scenario 1: all experimental treatments have the same effect as control in every biomarker subgroup**					
NLB	0.418	0.056	0.051	0.056	0.050
LB	0.409	0.048	0.074	0.040	0.040
PT	0.498	0.069	0.069	0.071	0.065
ER	0.473	0.067	0.068	0.069	0.069
**Scenario 2: as scenario 1, except T1 doubles the probability of response from 30% to 60% in patients who are positive for B1**					
NLB	0.856	0.051	0.767	0.058	0.057
LB	0.885	0.048	0.814	0.045	0.037
PT	0.903	0.070	0.817	0.057	0.068
ER	0.800	0.066	0.665	0.065	0.067
**Scenario 3: as scenario 1, except T1 doubles the probability of response in patients who are positive for B2**					
NLB	0.829	0.056	0.059	0.740	0.058
LB	0.790	0.054	0.062	0.676	0.038
PT	0.672	0.066	0.070	0.407	0.073
ER	0.802	0.066	0.067	0.666	0.067
**Scenario 4: T1 provides a moderate benefit in all biomarker groups**					
NLB	0.916	0.606	0.403	0.430	0.057
LB	0.912	0.600	0.503	0.356	0.032
PT	0.877	0.518	0.474	0.243	0.077
ER	0.869	0.526	0.367	0.362	0.070
**Scenario 5: T1 provides a detrimental effect in patients positive for B1 with response rate falling from 0.3 to 0.11**					
NLB	0.428	0.061	0.000	0.059	0.066
LB	0.367	0.047	0.000	0.033	0.048
PT	0.475	0.066	0.000	0.056	0.068
ER	0.440	0.070	0.001	0.064	0.065
**Scenario 6: as scenario 5, except T1 provides a detrimental effect in patients positive for B2 instead of B1**					
NLB	0.404	0.047	0.050	0.001	0.053
LB	0.406	0.051	0.073	0.000	0.044
PT	0.491	0.064	0.072	0.004	0.065
ER	0.450	0.070	0.070	0.001	0.069
**Scenario 7: T1 provides large benefit in patients positive for B1 and a detrimental effect in patients positive for B2**					
NLB	0.817	0.049	0.725	0.000	0.056
LB	0.878	0.057	0.806	0.000	0.033
PT	0.889	0.068	0.805	0.002	0.069
ER	0.787	0.070	0.657	0.002	0.069
**Scenario 8: T1 provides detrimental effect in patients positive for B1 and T2 provides a beneficial effect in those patients**					
NLB	0.848	0.056	0.002	0.046	0.774
LB	0.820	0.044	0.002	0.028	0.724
PT	0.662	0.071	0.001	0.063	0.412
ER	0.797	0.074	0.002	0.069	0.663

PT=parallel trials design; NLB=non-linked BAR design; LB=linked-BAR design; ER=equal-randomisation design.

Second to sixth columns give recommendation probabilities.
