# Supplementary material for: A Bayesian adaptive design for biomarker trials with linked treatments
Source: Br J Cancer. 2015 Aug 11;113(5):699–705. doi: 10.1038/bjc.2015.278 (PMC4559835; doi:10.1038/bjc.2015.278)

**Supplementary material for ‘A Bayesian adaptive design for biomarker trials with linked treatments’**

**Supplementary Materials and Methods**

**Notation**

A maximum number of N patients are recruited during the trial. The trial has a total of $J-1$ interim analyses, with a final analysis after all patients have been assessed. The jth interim analysis occurs after n_j_ patients have been recruited. We denote the number of experimental treatments by K, and assume there is also a control treatment. Each experimental treatment is linked with a biomarker, so that a priori it is thought plausible that patients who are positive for that biomarker are likely to benefit from the linked therapy. There are therefore a total of K biomarkers.

At recruitment, a patient is tested to determine which biomarkers they are positive for. We represent the biomarker profile for patient i by the vector $x_{i}=(x_{i1},\ldots,x_{iK})$. Each of the entries of $x_{i}$ takes the value 1 or 0, with $x_{ik}=1$ representing that patient i is positive for biomarker k. After testing, the patient is then assigned to treatment through one of the three procedures discussed later. The treatment received by patient i is labelled $t_{i}$, which takes a value in $\{0,1,\ldots,K\}$ (0 represents the control treatment). We define a K dimensional vector $T_{i}$ such that $T_{ik}=1$ if patient i is allocated to the kth experimental treatment, and 0 otherwise.

We assume, as in the motivating example, that the primary endpoint is binary, although similar techniques could be applied if the outcome was normally distributed. The response to treatment, i.e. whether or not the patient is a treatment success, is labelled $Y_{i}$. The value of $Y_{i}$ is 1 if a success is observed, and 0 otherwise.

**Interim analyses and treatment allocation**

The purpose of the interim analyses is to use the data gathered during the trial to update the allocation probabilities using a BAR procedure. We assume that the interim analyses are planned to occur when a certain number of patients have been recruited. At each interim analysis, a Bayesian logistic regression model is fitted that models the probability of treatment success as a function of treatment assignment, biomarker profile, and interactions between biomarker and treatment. The model for the probability of pCR for patient i is as follows:

$$\begin{matrix} Y_{i}\sim Bin(1,p_{i}) \\ \log\left(\frac{p_{i}}{1-p_{i}}\right)=\alpha+\sum_{k=1}^{K} T_{ik}\beta_{k}+\sum_{l=1}^{K} x_{il}\gamma_{l}+\sum_{k=1}^{K} \sum_{l=1}^{K} T_{ik}x_{il}\delta_{kl}, \end{matrix}$$

|  |  |  |  |
| --- | --- | --- | --- |

where $\alpha$ is the intercept term (or equivalently the log-odds of treatment success for a patient allocated to control who is positive for no biomarkers), $\beta_{k}$ is the main effect of experimental treatment k, $\gamma_{l}$ is the main effect of biomarker l, and $\delta_{kl}$ is the statistical interaction between experimental treatment k and biomarker l.

As we use a Bayesian approach, prior distributions are specified for all parameters. For $\alpha$, all $\beta$ parameters, and all $\gamma$ parameters, independent $U(-10,10)$ priors are specified. These are used as they are relatively uninformative. We also considered changing this prior to U(-2,2), but it made no discernible impact on the power of the trial approaches. We consider two possible specifications for the design prior (note that the final analysis does not use informative priors) of the $\delta$ parameters:

1. Similar to the other parameters, independent $U(-10,10)$ parameters are used for the $\delta$ parameters.
2. The diagonal entries of the $\delta$ matrix are given informative $N(1,1)$ priors. These diagonal entries correspond to the interaction parameters between the biomarkers and their linked treatment. Other entries of δ are given $U(-10,10)$ prior distributions.

The second case, with informative priors, is specified so that linked experimental treatments will be favoured at the interim analyses unless there is considerable evidence that an alternative treatment is superior. The specific value for the mean is chosen based on a sensitivity analysis presented later on.

For the first stage, allocation of a patient with biomarker profile $x_{i}$ is set to either be: 1) equal amongst all treatments or 2) equal amongst all linked treatments for their biomarker profile (or all treatments for patients who are negative for all biomarkers). Two BAR designs are considered: 1) the *non-linked BAR design*, which combines the non-informative δ prior distributions, and the initial allocation being open to all treatments; and 2) the *linked BAR design* which uses informative diagonal $\delta$ parameters, and starts by allocating patients only to control or to linked treatments.

The allocation probabilities after the first interim analysis are based on the posterior probabilities that each experimental treatment is superior to control. These posterior probabilities are calculated from equation 1 for each possible biomarker profile separately. For a given biomarker profile, x_i_, the posterior probability of treatment k being superior to control is:

Equation is used to update the allocation probabilities to each arm using a generalised version of the method used in Wason and Trippa (1). The allocation is set separately for every possible biomarker profile. The posterior probabilities of superiority $(\pi_{1}(x_{i}),\ldots,\pi_{k}(x_{i}))$ are calculated from equation . The allocation probabilities for a patient with biomarker profile $x_{i}, (p_{0}(x_{i}),p_{1}(x_{i}),\ldots,p_{k}(x_{i}))$ are set according to the following formula:

$$p_{k}(x_{i})\propto\left\{\begin{matrix} \frac{\pi_{k}(x_{i})^{\nu(n/N)}}{\sum_{k=1}^{K} \pi_{k}(x_{i})^{\nu(n/N)}} & \text{if }k=1,\ldots,K; \\ \frac{1}{K}\exp(\text{max}(n(x_{i})_{1},n(x_{i})_{2},\ldots,n(x_{i})_{K})-n(x_{i})_{0})^{\eta(n/N)} & \text{if }k=0; \end{matrix}\right.$$

where $n(x_{i})_{k}$ is the number of patients with biomarker profile $x_{i}$ recruited to arm k, and n is the total number of patients recruited to the trial so far. As in Wason and Trippa (1), $\nu$and $\eta$ are functions to modify the allocation as the trial progresses - we set them to $\eta(z)=\frac{1}{K}z$ and $\nu(z)=az^{b}$, where a and b are pre-specified to maximise the power of the trial. Since the allocation probabilities provided in do not generally sum to 1, they are then normalised.

Given the posterior probability of each arm being better than control that was calculated at the most recent interim analysis, the allocation only depends on the number of patients recruited to each arm and in particular does not require knowledge of the patient outcomes. Thus the allocation probability can be updated after each patient is recruited with a similar amount of administrative effort as a traditional RCT using stratified randomisation. Note however that the posterior probabilities in (2) are only updated at each interim analysis.

**Final analysis and hypothesis testing**

After all patients have been recruited and assessed, a final analysis occurs. A similar model to the one used at interim analyses (in equation ) is used, except fitted using a frequentist logistic regression instead of a Bayesian version. This is so that only data gathered in the trial are used to test the hypotheses. The prior distributions are only used to guide the adaptation, and not for the final analysis.

There are a total of K(K + 1) hypotheses that may be tested. A total of K correspond to the effect of an experimental treatment on patients who are positive for its linked biomarker. A further K(K - 1) will correspond to the effect of experimental treatments on patients who are positive for non-linked biomarkers. The remaining K hypotheses correspond to the effect of each experimental treatment on patients who are negative for every biomarker.

The complete set of null hypotheses tested are $\{H_{0}^{(k,l)},k=1,\ldots,K;l=0,\ldots,K\}$. For l > 0, $H_{0}^{(k,l)}$ is the null hypothesis that experimental treatment k provides no benefit over control in patients who are positive for biomarker l. The hypothesis $H_{0}^{(k,0)}$ is that experimental treatment k provides no benefit over control in patients who are negative for all biomarkers. In other words:

$$H_{0}^{(k,l)}:\left\{\begin{matrix} \beta_{k}+\delta_{kl}\leq0 & \text{if }l>0 \\ \beta_{k}\leq0 & \text{if }l=0 \end{matrix}\right.$$

One could also test hypothesis of experimental treatment benefit in patients positive for specific sets of biomarkers, but we do not consider this here. As there are multiple null hypotheses, the total probability of making a type-I error is higher than the significance level used for each null hypothesis. This total probability of type-I error is known as the family-wise error rate (FWER). As we are considering a phase II setting where significant results will be tested in an appropriately powered phase III trial, we do not aim to control the FWER at a stringent rate. Instead we set a critical test statistic value to control the FWER at around 0.4-0.5. Although this seems high, previous work has shown that high FWERs can be optimal when considering multi-arm phase II trials (2). Note also that the FWER will not be as high as the situation where K(K + 1) independent null hypotheses are being tested, as there is considerable overlap between the parameters making up each null hypothesis for a given experimental treatment (e.g. $\beta_{k}$ appears in each null hypothesis associated with treatment k).

Any null hypotheses that are rejected will result in consideration of a phase III trial in the relevant sub-population. For example, if $H_{0}^{(1,1)}$ is rejected, then a phase III trial of experimental treatment 1 versus control in patients who are positive for biomarker 1 will be considered. If multiple null hypotheses are rejected, then it may be than multiple subgroups are considered in the same phase III trial. We note that hypothesis testing results in this trial are non-binding and that other factors will be considered before starting a phase III trial.

**Supplementary results**

**Technical description of simulation scenarios**

Table 1 provides a more technical description of the simulation scenarios considered in the main paper.

Table 1 - Description of simulation scenarios. In all cases, prevalence of each biomarker is set to 0.3.

| Scenario number | Scenario description |
| --- | --- |
| 1 | All $\beta$, $\gamma$ and $\delta$ set to 0, $\alpha$ is set to -0.85. |
| 2 | As scenario 1, except $\delta_{11}=1.25.$ |
| 3 | As scenario 1, except $\delta_{12}=1.25.$ |
| 4 | As scenario 1, except $\beta_{1}=0.75.$ |
| 5 | As scenario 1, except $\delta_{11}=-1.25.$ |
| 6 | As scenario 1, except $\delta_{12}=-1.25.$ |
| 7 | As scenario 1, except $\delta_{11}=1.25,\delta_{12}=-1.25.$ |
| 8 | As scenario 1, except $\delta_{11}=-1,\delta_{21}=1.25.$ |

**Varying number of interim analyses**

In the main paper we consider designs with five stages (four interim analyses and a final analysis). Previous work (1), in the context of multi-arm trials without biomarker subgroups, showed that five stages was broadly the correct number to balance power and logistical issues – going beyond five stages did not result in sufficient power gain to justify the additional analyses. It is of course possible that this is different when there are biomarker subgroups.

To address this, we conducted additional simulation studies where the number of analyses was varied between two and ten. The spacing of the interim analyses is shown in Table 2.

Table 2 – interim analysis spacing as the number of stages/analyses changes.

| Number of stages | Spacing |
| --- | --- |
| 2 | (175, 350) |
| 3 | (116, 233, 350) |
| 4 | (100, 183, 267, 350) |
| 5 | (100, 162, 225, 287, 350) |
| 6 | (100, 150, 200, 250, 300, 350) |
| 7 | (100, 141, 183, 224, 266, 307, 350) |
| 8 | (100, 135, 171, 206, 242, 278, 313, 350) |
| 9 | (100, 131, 162, 193, 225, 256, 287, 318, 350) |
| 10 | (100, 127, 155, 183, 210, 238, 265, 293, 321, 350) |

At each analysis, the allocation was updated according to the results so far. A recruitment rate of 7 patients per month was assumed. For each simulation, 2500 simulation replicates were used.

Figure 1 shows the power of the linked-BAR method to detect a significant difference between T1 and control as the number of stages varies. Two scenarios are considered, corresponding to scenarios 2 and 3 in the main simulation study in the paper. The power is shown for B1-positive patients in scenario 2 and B2-positive patients in scenario 3.

Figure 1 shows the number of stages makes little difference to the power in scenario 2. However there is evidence that the power in scenario 3 depends on the number of stages. A minimum of four stages is required – going beyond five stages provides little or no advantage to the power.

**Varying the prior mean used for interaction parameters**

The linked-BAR design uses informative priors for the interaction terms corresponding to linked biomarker-treatment pairs (i.e. $\delta_{1},\delta_{2},\delta_{3}$). In the main paper we use a prior variance of 1. We carried out simulation studies to explore which prior mean was the best value to use.

We again considered scenarios 2 and 3 from the main paper, and varied the prior mean from 0 to 3 in increments of 0.25. The five stage design from the main paper is considered, with 2500 replicates per simulation scenario. Figure 2 shows these results.

Interestingly, figure 2 shows that larger values of the prior mean make little difference to the power under scenario 2, but do result in moderate power loss under scenario 3. Given these results, we used a prior mean of 1 in the main paper.

Reference List

(1) Wason J, Trippa L. A comparison of Bayesian adaptive randomization and multi-stage designs for multi-arm clinical trials. *Statist Med* 2014,**Epublished ahead of print**.

(2) Wason JMS, Jaki T, Stallard N. Planning multi-arm screening studies within the context of a drug development program. *Statist Med* 2013 Sep 10,**32**(20), 3424-3435.

Figure 1 – power as the number of interim analyses changes. The interim analysis spacing is given in table 2. Scenario 2 – T1 provides a treatment benefit for B1-positive patients only; scenario 3 – T1 provides a treatment benefit for B2-positive patients only.


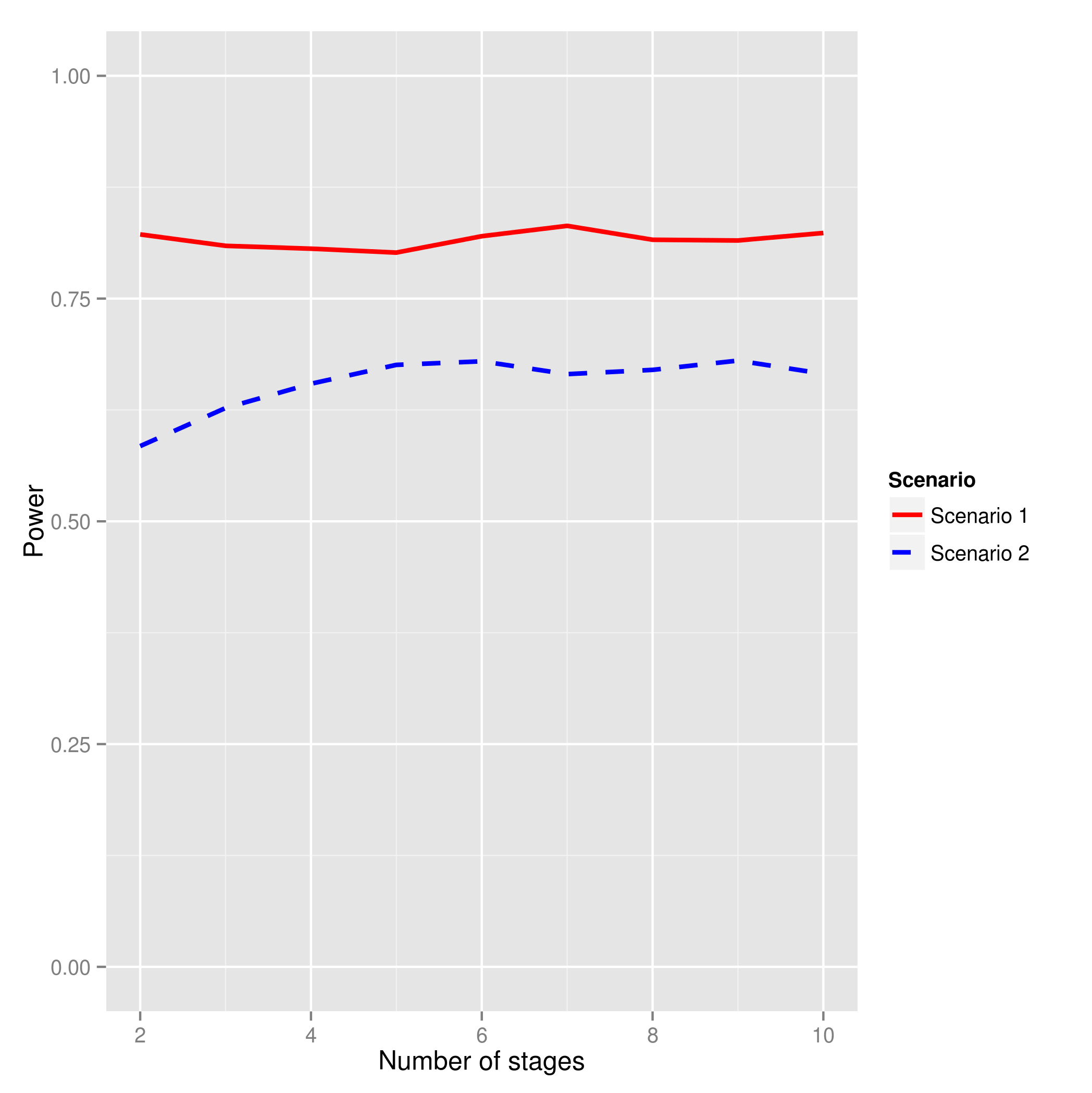


Figure 2 – power as the prior mean used for the interaction parameters between linked treatments and biomarkers. Scenario 2 – T1 provides a treatment benefit for B1-positive patients only; scenario 3 – T1 provides a treatment benefit for B2-positive patients only.


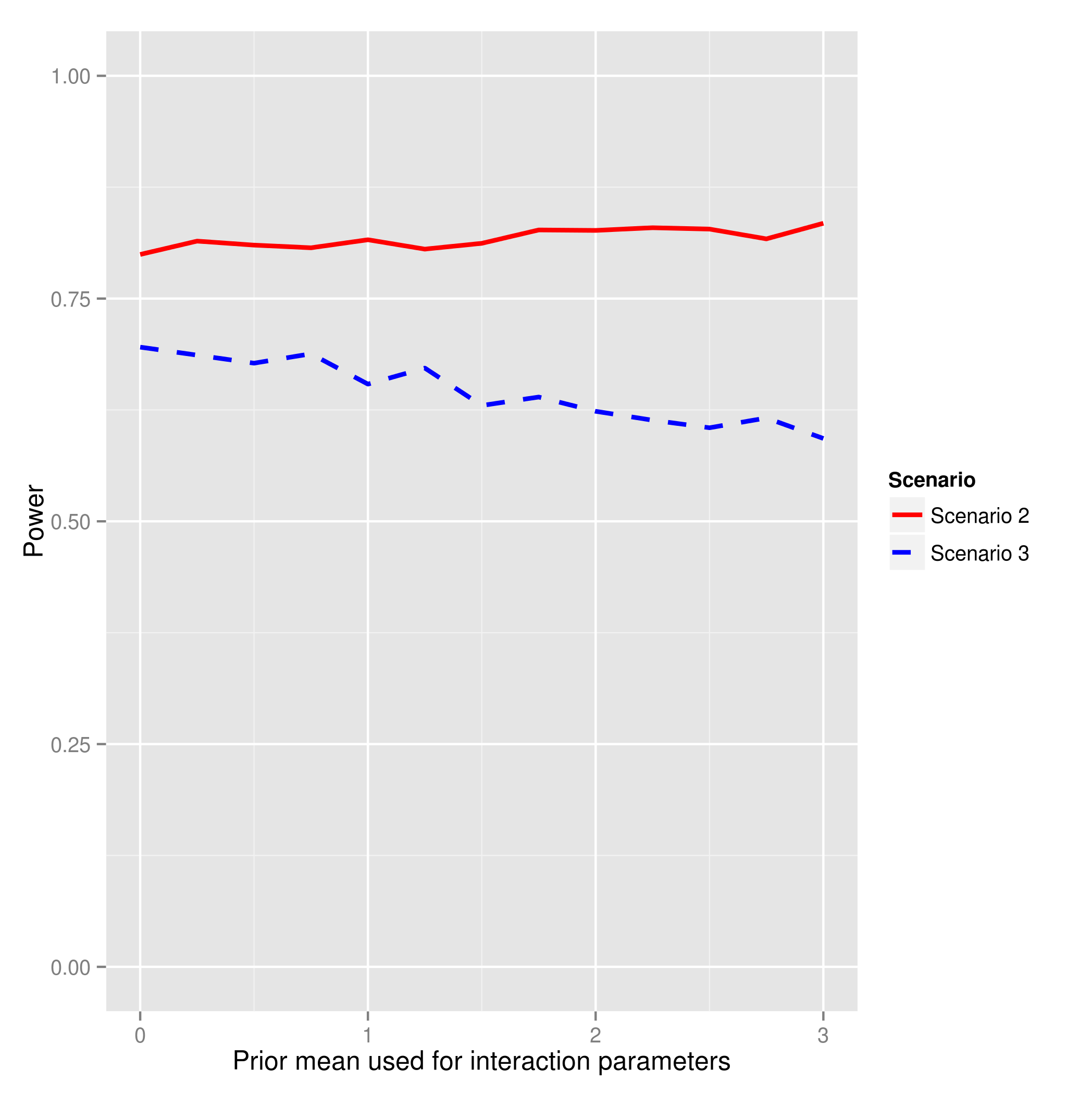

Supplement: Supplementary Information [file bjc2015278x1.docx]
